# Outcome and survival analysis of surgical repair of post-infarction ventricular septal rupture

**DOI:** 10.1186/1749-8090-8-44

**Published:** 2013-03-09

**Authors:** Philip YK Pang, Yoong Kong Sin, Chong Hee Lim, Teing Ee Tan, See Lim Lim, Victor TT Chao, Jang Wen Su, Yeow Leng Chua

**Affiliations:** 1 Department of Cardiothoracic Surgery National Heart Centre, Mistri Wing 17 Third Hospital Avenue, Singapore, 168752, Singapore

**Keywords:** Ventricular septal rupture, Myocardial infarction

## Abstract

**Background:**

To review the experience of surgical repair of post-infarction ventricular septal rupture (VSR) and analyze the associated outcomes and prognostic factors.

**Methods:**

Following approval from the Singhealth Centralised Institutional Review Board (reference: 2011/881/C), a retrospective review was performed on 38 consecutive patients who had undergone surgical repair of post-infarction VSR between 1999 and 2011. Continuous variables were expressed as either mean ± standard deviation or median with 25^th^ and 75^th^ percentiles. These were compared using two-tailed t-test or Mann–Whitney U test respectively. Categorical variables were compared using chi-square or Fisher’s exact test. To identify predictors of operative mortality, univariate analysis of perioperative variables followed by multivariate analysis of significant univariate risk factors was performed. A two-tailed p-value < 0.05 was used to indicate statistical significance.

**Results:**

Mean age was 65.7 ± 9.4 years with 52.6% males. The VSR was anterior in 28 (73.7%) and posterior in 10 patients. Median interval from myocardial infarction to VSR was 1 day (1, 4). Pre-operative intra-aortic balloon pump was inserted in 37 patients (97.8%). Thirty-six patients (94.7%) underwent coronary angiography.

Thirty-five patients (92.1%) underwent patch repair. Mean aortic cross clamp time was 82 ± 40 minutes and mean cardiopulmonary bypass time was 152 ± 52 minutes. Coronary artery bypass grafting (CABG) was performed in 19 patients (50%), with a mean of 1.5 ± 0.7 distal anastomoses. Operative mortality within 30 days was 39.5%.

Univariate analysis identified emergency surgery, New York Heart Association (NYHA) class, inotropic support, right ventricular dysfunction, EuroSCORE II, intra-operative red cell transfusion, post-operative renal failure and renal replacement therapy (RRT) as predictors of operative mortality. Multivariate analysis identified NYHA class and post-operative RRT as predictors of operative mortality.

Ten year overall survival was 44.4 ± 8.4%. Right ventricular dysfunction, LVEF and NYHA class at presentation were independent factors affecting long-term survival. Concomitant CABG did not influence early or late survival.

**Conclusions:**

Surgical repair of post-infarction VSR carries a high operative mortality. NYHA class at presentation and post-operative RRT are predictors of early mortality. Right ventricular dysfunction, LVEF and NYHA class at presentation affect long-term survival. Concomitant CABG does not improve survival.

## Background

The reported incidence of acute ventricular septal rupture (VSR) following acute myocardial infarction (AMI) is 1% to 3% in the era prior to widespread reperfusion therapy [1]. The incidence has since declined to around 0.3% following the advent of thrombolysis [2].

Despite advances in critical care and prompt surgical intervention, the incidence of post-infarction VSR has not changed during the past 2 decades. The mortality rate of post-infarction VSR remains high and relatively constant [3]. Reported 30-day mortality rates range from 19 to 54% [3-11].

This study aims to investigate the survival outcome and prognostic factors associated with surgical repair of post-infarction VSR at a tertiary referral centre over a 13 year period.

## Methods

A retrospective case-note and database review was performed on consecutive patients who had undergone surgical repair of post-infarction VSR between January 1999 and December 2011 at our tertiary referral centre. This study was approved by the Singhealth Centralised Institutional Review Board (reference: 2011/881/C).

### Definitions

Early or operative mortality was defined as death within 30 days of surgery, either in hospital or after hospital discharge. Renal failure was defined by serum creatinine levels higher than 1.36 mg/dl or the need for renal replacement therapy (RRT). Inotropic support was defined as infusions of dopamine over 5 mcg/kg/min or any use of adrenaline, noradrenaline or vasopressin. Emergency surgery was defined as surgery within 24 hours of diagnosis of VSR. Post-operative RRT was in the form of Continuous Veno-Venous Haemofiltration (CVVH) or Continuous Veno-Venous Haemodiafiltration (CVVHDF).

### Patients

During the period January 1999 to December 2011, 42 consecutive patients were referred to our tertiary cardiac centre following diagnosis of VSR. Four patients with anterior VSRs underwent medical therapy as 2 patients declined surgery and the other 2 were not offered surgery in view of their advanced age (both 91 years old), multiple medical comorbidities and poor premorbid functional status. All 4 patients died within 5 days of diagnosis of VSR. Thirty eight patients underwent surgical repair of VSR. No patients were treated with percutaneous closure devices.

Data was retrospectively collected from patient case notes and electronic records. Patient demographics and comorbidities are shown in Table 1. All patients were followed-up at our institution, with 1 patient lost to follow-up. Male patients accounted for 52.6%. Mean age was 65.7 ± 9.4 years (range 45 – 83). The mean age of females was significantly higher than males (70.0 ± 6.0 vs 61.8 ± 10.3 years, *P*=0.005). In all patients, a new cardiac murmur was present in the setting of an ST-segment elevation myocardial infarction (STEMI). Transthoracic echocardiography (TTE) was performed for all patients to confirm the diagnosis of VSR and exclude the differential diagnosis of acute mitral regurgitation from papillary muscle rupture. Evidence of right ventricular (RV) dysfunction such as RV dilatation or wall motion abnormality was also diagnosed with pre-operative echocardiography. RV dysfunction was present in 10 patients (26.3%), of which 7 had presented with inferior MI and 3 with anterior MI. RV dysfunction was present in 8 of 10 (80%) with posterior VSRs and 2 of 28 (7.1%) with anterior VSRs, (P<0.0005). The VSR location was anterior in 28 patients (73.7%) and posterior in 10 patients (26.3%). One patient had a concomitant ventricular free wall rupture. The median time interval between myocardial infarction and septal rupture was 1 day (1, 4). The interval between myocardial infarction and septal rupture was 1 day in 20 patients (52.5%), 2 to 7 days in 13 patients (37.0%) and 8 to 13 days in 4 patients (10.5%).

**Table 1:** Patient demographics and comorbidities

**Variable**	**Survivors** **n = 23 (%)**	**Non-survivors** **n = 15 (%)**	***P*-value**
**Demographics**			
Age (years)	64.7 ± 9.2	67.3 ± 9.9	0.406
Gender (Male)	11 (47.8)	9 (60.0)	0.463
BMI (kg/m^2^)	25.1 ± 3.6	23.7 ± 2.0	0.186
LVEF (%)	40.3 ± 11.0	39.1 ± 9.4	0.746
**Comorbidities**			
Smoking	8 (34.8)	8 (53.3)	0.258
Diabetes mellitus	8 (34.8)	4 (26.7)	0.599
Renal failure	9 (39.1)	6 (40.0)	0.957
Hypertension	16 (69.6)	10 (66.7)	0.851
Hyperlipidaemia	9 (39.1)	5 (33.3)	0.717
Previous stroke	2 (8.7)	2 (13.3)	0.649

BMI: Body Mass Index.

LVEF: Left Ventricular Ejection Fraction.

**Figure 1:**
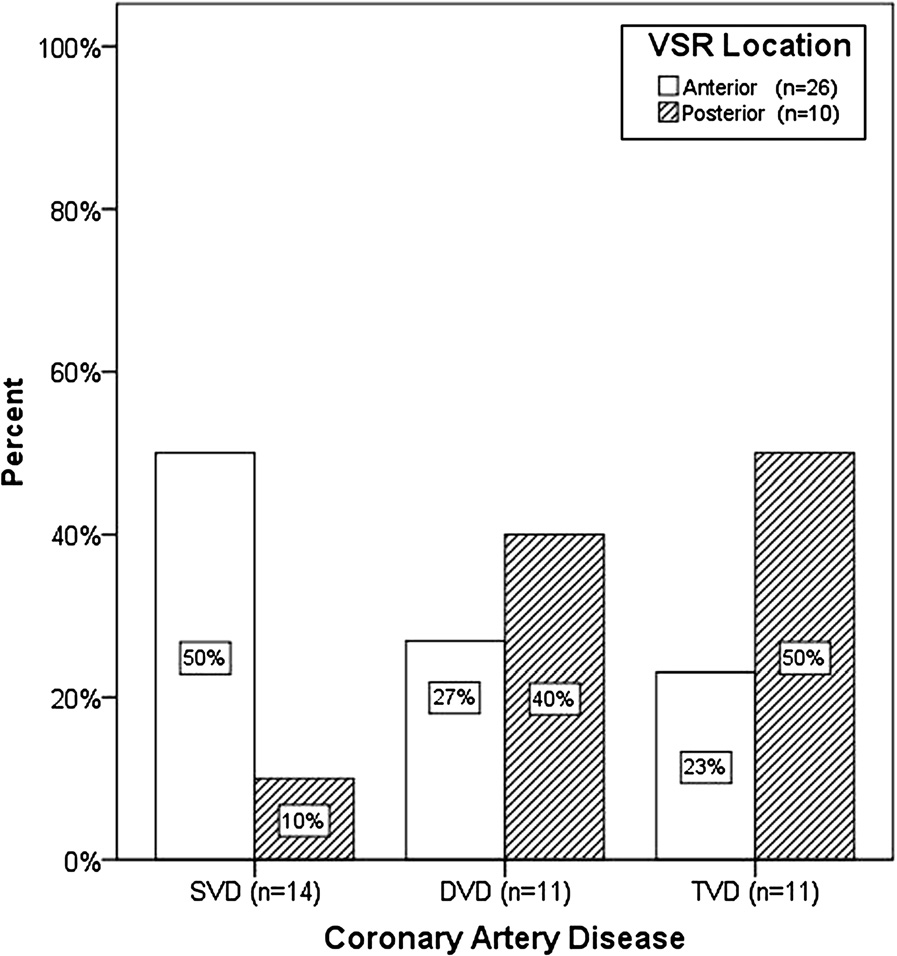
**Distribution of coronary artery disease according to VSR location.** DVD: Double vessel disease SVD: Single vessel disease TVD: Triple vessel disease VSR: Ventricular septal rupture.

Eleven patients (28.9%) had undergone thrombolysis with either streptokinase or recombinant tissue plasminogen activator. Coronary angiography was performed in 36 patients (94.7%), which showed single vessel disease (SVD) in 38.8%, double vessel disease (DVD) in 30.6% and triple vessel disease (TVD) in 30.6% of patients. Within the SVD group, 10 of 14 patients (71.4%) had total occlusion of the infarcted artery. Figure 1 shows the distribution of coronary disease according to VSR location.

### Statistical analysis

Statistical analysis was performed for the surgically treated group, using SPSS version 17 statistical software. Continuous variables were expressed as either mean ± standard deviation or median with 25^th^ and 75^th^ percentiles, depending on the normality of distribution. These were compared using two-tailed t-test or Mann–Whitney U test respectively. Categorical variables, expressed as percentages, were analysed with chi-square or Fisher’s exact test. To identify risk factors predictive of operative mortality, univariate analysis of perioperative variables was performed. Significant univariate risk factors were examined using backward logistic regression analysis. Survival function was presented using Kaplan-Meier survival curves and comparisons performed with log-rank test. A multivariate Cox proportional hazard analysis was performed to identify independent factors affecting long-term survival. A two-tailed p-value less than 0.05 was used to indicate statistical significance.

## Results

### Operative technique

Thirty-seven operations (97.4%) were performed via median sternotomy. One patient underwent VSR repair through a left anterior thoracotomy. Moderately hypothermic cardiopulmonary bypass was instituted in all cases. Aortic cross clamping and cold cardioplegic myocardial protection was applied in 34 patients (89.5%). Mean cardiopulmonary bypass time was 152 ± 52 minutes and mean aortic cross clamp time was 82 ± 40 minutes.

Single patch closure was performed in 35 cases (92.1%), using either a Dacron or bovine pericardial patch, adopting an infarct exclusion technique similar to that of David *et al*. [8]. Two cases were repaired by direct suturing without a patch. In 1 case, the VSR was too extensive and not amenable for repair by any method, resulting in an immediate post-operative death. The ventriculotomy was closed by direct suture buttressed on felt strips in all patients. Two patients underwent tricuspid valve repair and one patient required implantation of a left ventricular assist device due to inability to wean off cardiopulmonary bypass. Coronary artery bypass grafting was performed in 19 patients (50.0%), with a mean of 1.5 ± 0.7 distal anastomoses.

### Early outcome

Emergency surgery was performed for 32 patients (84.2%). An intra-aortic balloon pump (IABP) was inserted in 37 patients (97.4%) prior to surgery. The median time interval between the diagnosis of VSR and surgery was 9 hours (6, 30). Thirty-six patients (94.7%) underwent VSR repair within 5 days of diagnosis. Two patients were stabilized with medical therapy and underwent VSR repair 28 and 82 days after diagnosis respectively.

Fifteen patients died within 30 days of operation, giving an operative mortality of 39.5%. There was 1 intra-operative mortality. There was no significant difference in the duration of aortic cross clamping between survivors and non-survivors, (90 ± 27 minutes vs 93 ± 34 minutes respectively, *P*=0.742). Table 2 shows the univariate risk analysis for predictors of operative mortality. SVD was more common in anterior VSRs compared to posterior VSRs (50.0% vs 10.0%, *P*=0.027).

**Table 2:** Univariate risk analysis for operative mortality

**Variable**	**Survivors** **n = 23 (%)**	**Non-survivors** **n = 15 (%)**	***P*-value**
**Pre-operative**			
NYHA Class (II-III / IV)	12 (52.2) / 11 (47.8)	1 (6.7) / 14 (93.3)	0.004
Emergency Surgery	17 (73.9)	15 (100)	0.031
Cardiogenic Shock	13 (56.5)	13 (86.7)	0.051
Inotropic support	7 (30.4)	10 (66.7)	0.028
Type of MI (Anterior/Inferior)	19 (82.6) / 4 (17.4)	10 (66.7) / 5 (33.3)	0.259
Right ventricular dysfunction	3 (13.0)	7 (46.7)	0.021
Multi-vessel coronary artery disease	13 (56.5)	9 (69.2)	0.452
Total occlusion of infarcted artery	16 (69.6)	10 (76.9)	0.636
Thrombolysis	7 (30.4)	4 (26.7)	0.802
Intra-aortic balloon pump	22 (95.7)	15 (100)	0.413
EuroSCORE II	22.8 ± 9.4	34.3 ± 9.8	0.001
**Intra-operative**			
VSR Location (Anterior/Posterior)	19 (82.6) / 4 (17.2)	9 (60.0) / 6 (40.0)	0.122
CPB time (minutes)	148.0 ± 52.3	158.8 ± 53.0	0.540
Red blood cell transfusion > 2 units	4 (17.4)	8 (57.1)	0.012
Concomitant CABG	12 (52.2)	7 (46.7)	0.740
**Post-operative**			
Renal failure	8 (34.8)	11 (73.3)	0.020
Renal replacement therapy	2 (8.7)	10 (66.7)	<0.0005
Re-exploration for bleeding	4 (17.2)	4 (26.7)	0.493
DIVC	0 (0)	3 (20.0)	0.025

CABG: Coronary artery bypass grafting.

CPB: Cardiopulmonary bypass.

DIVC: Disseminated intravascular coagulopathy.

EuroSCORE: European System for Cardiac Operative Risk Evaluation II.

MI: Myocardial infarction.

NYHA: New York Heart Association.

VSR: Ventricular septal rupture.

Pre-existing medical conditions did not significantly affect early mortality. Significant predictors of operative mortality identified using univariate analysis included emergency operation, NYHA class, inotropic support, right ventricular dysfunction, EuroSCORE II, intra-operative red blood cell transfusion, post-operative renal failure and RRT. Logistic regression for multivariate analysis showed that the NYHA class at presentation and post-operative RRT were independent risk factors for operative mortality, as shown in Table 3.

**Table 3:** Logistic regression for operative mortality

**Variable**	**Odds ratio (OR)**	**95% confidence interval**	**p-value**
Post-operative RRT	26.4	2.7 – 260.9	0.005
NYHA Class IV	20.3	1.3 – 320.0	0.033

NYHA: New York Heart Association.

RRT: Renal replacement therapy.

Post-operative echocardiography diagnosed a residual shunt of various degrees in 9 patients (23.7%). One patient had a near complete patch dehiscence requiring early re-operation on the 8^th^ post-operative day and subsequently succumbed to multi-organ failure. The other 8 patients with less severe shunts were managed conservatively.

### Late outcome

**Figure 2:**
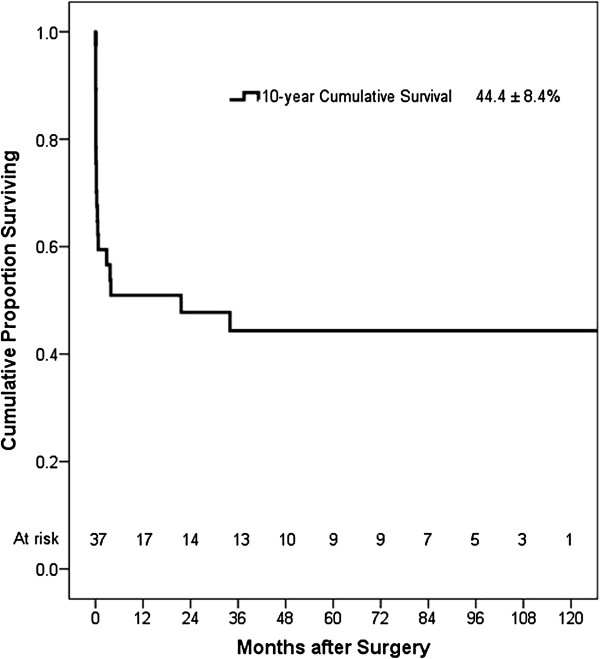
Kaplan-Meier estimate of overall survival, including operative deaths.

Follow-up was 97.4% complete with one patient lost to follow-up. Data regarding follow-up was obtained by direct assessment during scheduled clinic reviews at our institution. There were 5 late deaths due to cardiac causes. Of these, 3 patients died from congestive cardiac failure and 2 patients died from recurrent myocardial infarction, occurring at a median interval of 3.9 months (3.2 months, 27.8 months) following VSR repair. Three patients died from non-cardiac related causes. Figure 2 shows the Kaplan-Meier estimate of overall survival, including operative deaths. Overall survival at 10 years was 44.4 ± 8.4%.

Twenty-three patients (60.5%) survived to hospital discharge. The median follow-up period was 41.1 months (18.2 months, 96.5 months). At latest follow-up, 12 patients (52.2%) were in NYHA class I, 6 (26.1%) in class II, 3 (13.0%) in class III and 2 (8.7%) in class IV. Six patients (26.1%) in NYHA II to IV were being treated with at least one heart failure medication at latest follow-up. Sixteen patients (69.6%) were free from angina during follow-up. Five patients (21.7%) had Canadian Cardiovascular Society (CCS) grade I angina and 2 (8.7%) had grade II angina. All symptomatic patients received anti-anginal medication. Six patients who were employed prior to VSR surgery were all able to resume work following recovery.

**Figure 3:**
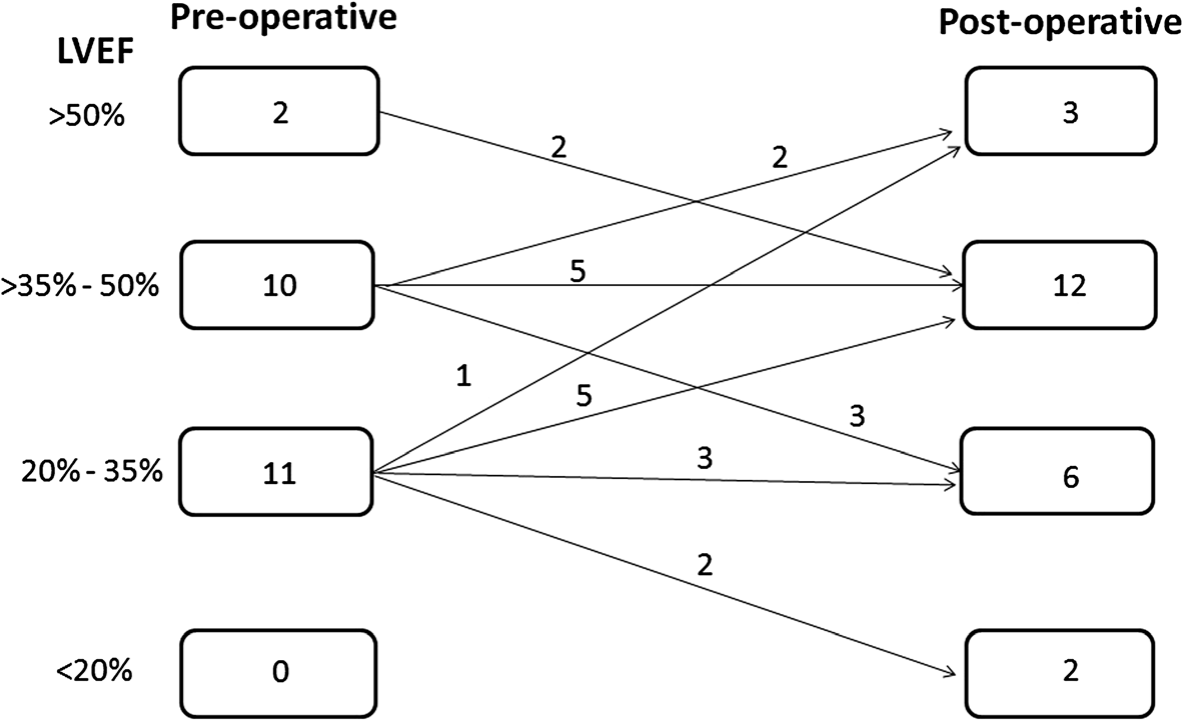
**Distribution of patients grouped according to LVEF on follow-up.** LVEF: Left ventricular ejection fraction.

Post-discharge echocardiography was performed at a median interval of 5.9 months (1.4 months, 17.8 months) after VSR repair. Figure 3 shows the distribution of patients grouped according to LVEF on follow-up. Nine patients (39.1%) had LVEF ≤35% during follow-up. None of these patients received implantable cardiac defibrillators.

Six patients with residual shunts survived to hospital discharge. These residual shunts ranged from 3 mm to 10 mm in size and were located anteriorly in seven patients (87.5%) and posteriorly in 1 patient (12.5%). Table 4 summarizes the characteristics and outcomes of patients with residual shunts. No patients developed new shunts during follow-up echocardiographic surveillance.

**Table 4:** Characteristics and outcomes of patients with residual shunts

		**Residual shunt**			**Follow-up**				
**Patient**	**Age**	**Location**	**Size (mm)**	**Progression**	**Shunt size (mm)**	**NYHA class**	**Outcome**	**Cause of death**	**Survival (months)**
1	46	Anterior	5	No	5	IV	Non-survivor	Multi-organ failure	0.7
2	57	Posterior	5	No	5	IV	Non-survivor	Congestive cardiac failure	2.8
3	73	Anterior	10	Yes	18	III	Non-survivor	Congestive cardiac failure	3.7
4	64	Anterior	5	No	5	II	Non-survivor	Recurrent myocardial infarction	22
5	69	Anterior	5	No	0	I	Non-survivor	Recurrent myocardial infarction	34
6	63	Anterior	4	No	4	I	Survivor		16
7	70	Anterior	5	No	5	I	Survivor		98
8	63	Anterior	3	Yes	7	I	Survivor		112

NYHA: New York Heart Association.

Table 5 shows the results of multivariate Cox regression analysis of factors affecting late survival. Factors identified as having a significant negative impact on late survival were NYHA Class IV at the time of presentation, RV dysfunction and lower LVEF.

**Table 5:** Multivariate Cox regression analysis for predictors of late cardiac-related mortality

**Variable**	**Hazard ratio (HR)**	**95% confidence Interval (CI)**	**p-value**
RV Dysfunction	8.5	1.2 – 60.1	0.033
NYHA Class IV	5.2	1.2 – 22.0	0.026
LVEF	0.93	0.87 – 0.99	0.022
CABG	0.52	0.13 – 2.12	0.360

CABG: Coronary artery bypass grafting.

LVEF: Left Ventricular Ejection Fraction.

NYHA: New York Heart Association.

RV: Right ventricular.

## Discussion

The availability of early reperfusion therapy for myocardial infarction has led to the declining incidence of post-infarction VSRs. Despite this trend, VSR remains one of the most challenging conditions encountered by cardiac surgeons today.

The 30-day operative mortality in this series was 39.5%, similar to the rates of 34% to 37% reported in other series [4-7]. Lower operative mortality rates ranging from 19% to 23% have been reported [8-11]. Univariate analysis identified emergency operation as a predictor of early mortality in this series, which was similar to the findings of other authors [4,6,9,10]. The same authors also identified the duration of aortic cross clamp as a predictor of early mortality but this association was not demonstrated in this study. Increased cardiopulmonary bypass time has also been reported as a predictor of operative mortality but this association was not evident in this series [11-14].

The optimal timing of surgical repair for VSRs is critical. A longer interval before surgery has been reported to be associated with improved survival [15]. Despite this, only 2 of our 38 patients remained sufficiently stable in NYHA class II to permit delayed surgery. Although both these patients survived with good outcomes and returned to NYHA class I after recovery, we reserve delayed VSR repair only for a carefully selected group of patients who remain haemodynamically stable.

We adopted the single patch technique in 35 cases (92.1%). The incidence of post-operative residual shunt was 23.7%, comparable to the incidence of 24 to 26% described in other series in which a variety of techniques were used [6,16]. An alternative double patch technique has been reported to decrease the incidence of residual shunt from 11% to 0% [17].

We routinely perform coronary angiography for all haemodynamically stable patients diagnosed with VSR at our centre. This provides an opportunity for the insertion of IABP under fluoroscopic guidance. In this series, coronary angiography was performed in all except for 2 patients who presented in extremis. SVD was significantly more common in anterior VSRs compared to posterior VSRs in this series, similar to the findings of Davies *et al.*[18]. In the group with SVD, 71.4% had total occlusion of the infarcted artery. Reported figures range from 57% to 82% [2,19].

Lundblad *et al.*[20] found that concomitant CABG during VSR repair reduces both early and late mortality when compared with patients with unbypassed coronary artery disease. Although there was no significant difference in early mortality within other series, mid to long-term survival benefit has been reported [16,20-22].

In a review of recent literature, Perotta *et al.*[23] reported an improvement of mortality rates from 26.3% in those without CABG to 21.2% in those who had undergone CABG. These results applied to patients with multi-vessel disease where complete myocardial revascularization was achieved by bypassing all stenotic coronary arteries supplying non-infarcted areas. Actuarial survival at five years from this series ranged from 29% to 72%. In this study, there was no statistically significant early or long-term survival advantage for patients who had undergone concomitant CABG. Other authors have reported similar findings [4,6,7,10,11,15,17,24]. Despite the lack of significant survival benefit associated with concomitant CABG in their studies, some authors advocate concomitant CABG during VSR repair as long as it can be performed safely [4,5,15,17]. The aim is to reduce further ischaemic risk associated with multi-vessel coronary artery disease by improving collateral flow to the myocardium.

Similar to the findings of Moore *et al*. [25], our data shows that RV dysfunction has a negative impact on early and late survival. As RV failure ensues, the left-sided cardiac chambers are unable to fill, leading to biventricular failure. This exacerbates the low cardiac output state, contributing towards a higher mortality rate.

Percutaneous closure has been increasingly used in patients with postinfarction VSRs, initially in patients with recurrent ventricular septal defects (VSDs) after primary surgical repair, but more recently as primary therapy in patients with acute VSR and high surgical risk, or as a temporizing bridge to surgery [26]. Ventricular septal occluder devices were initially developed for percutaneous closure of congenital VSDs. Percutaneous deployment of an occluding device may be precluded by the geometry and location of the VSR, or interference from valvular apparatus, especially in posterior VSRs. The highest technical success has been reported with the Amplatzer device (AGA Medical Corp, Plymouth, MN), with procedural success rates ranging from 86% to 89% [27,28]. Lee *et al*. [29] recently described the use of a hybrid approach with the advantage of allowing direct manipulation of the Amplatzer device. This approach prevents interference of the valve apparatus, avoiding the need to perform ventriculotomy in an already infarcted ventricle.

Large (≥15 mm) VSRs should undergo immediate surgery as these are prone to device embolization or residual VSR. Amplatzer closure can be used as definitive therapy for small or medium VSRs. It can also be used to stabilize patients and allow myocardial fibrosis, thus facilitating delayed surgical correction [27]. Despite a less invasive technique, procedural mortality and morbidity are high, especially in patients with cardiogenic shock (mortality in cardiogenic shock 88% vs. 38% non-shock P<0.001) [26]. Overall 30-day mortality rates for percutaneous closure range from 28% to 65% [26-28]. Other procedure-related major complications include residual shunting, left ventricular rupture and device embolization. The incidence of residual shunts ranges from 8.3% to 13.8%, much lower than 73% reported in an earlier series [26-28].

Ventricular assist devices (VADs) are useful adjuncts for the treatment of VSRs in the setting of univentricular or biventricular failure, either preoperatively as a bridge to surgery or postoperatively following VSR repair. Mechanical circulatory support with VADs allows restoration of peripheral organ perfusion and provides an opportunity for recovery and maturation of the infarcted myocardium before secondary VSR repair. Applying this concept, a staged approach of initial left ventricular or biventricular mechanical support with an implantable left ventricular assist device (LVAD) or biventricular assist device (BiVAD) followed by secondary VSR repair has been described [30,31]. Previously, BiVAD support without VSR repair has been described as a bridge-to-transplantation approach [32].

La Torre *et al*. [33] reported a similar bridge to surgery strategy in their series of patients with posterior VSRs using the Impella Recover (ABIOMED, Inc, Danvers, Mass), a percutaneous left ventricular assist device (LVAD). Utilizing an intravascular microaxial blood pump inserted in the femoral artery, successful reduction in left-to-right shunting and increase in cardiac output was achieved while awaiting VSR repair about 3 weeks later. Despite successful surgical repair of VSR, some patients with extensive infarction and persistent ventricular failure remain in cardiogenic shock and require VAD support to maintain physiologic haemodynamics. Implantable LVADs have been used successfully to bridge these patients to cardiac transplantation [34].

This is a retrospective descriptive study with inherent biases in data collection. Due to the relatively rare occurrence of post-infarction VSR, the small sample size underpowered the statistical analysis and could have limited the number of statistically significant variables. A prospective multi-centre study incorporating a larger sample size would be useful to assess the prognostic value of the risk factors identified.

## Conclusions

Post-infarction VSR remains a serious and challenging complication of acute myocardial infarction in the modern surgical era. Surgical repair is associated with an operative mortality of 39.5%. Overall survival at 10 years is 44.4% ± 8.4%. The NYHA class at presentation and the need for post-operative RRT are independent predictors of early mortality. Right ventricular dysfunction, lower LVEF and NYHA class IV symptoms at presentation are predictors of poor long-term survival. Concomitant CABG during VSR repair does not confer a significant survival advantage.

## Abbreviations

AMI: Acute myocardial infarction; BiVAD: Biventricular assist device; CABG: Coronary artery bypass grafting; CVVH: Continuous veno-venous haemofiltration; CVVHDF: Continuous veno-venous haemodiafiltration; DVD: Double vessel disease; IABP: Intra-aortic balloon pump; LVAD: Left ventricular assist device; NYHA: New York Heart Association; RRT: Renal replacement therapy; RV: Right ventricle; STEMI: ST-elevation myocardial infarction; SVD: Single vessel disease; TTE: Transthoracic echocardiography; TVD: Triple vessel disease; VSD: Ventricular septal defect; VAD: Ventricular assist device.

## Competing interests

The authors declare that they have no competing interests.

## Authors’ contributions

PPY designed the study, performed data collection, analysis and interpretation and drafted the article. SYK conceptualized the study, performed critical revisions and provided the final approval of the article. LCH assisted in data interpretation and performed critical revisions of the article. TTE performed critical revisions of the article. LSL performed critical revisions of the article CVT performed critical revisions of the article. SJW assisted in data interpretation and performed critical revisions of the article. CYL assisted in data interpretation and performed critical revisions of the article. All authors read and approved the final manuscript.
